# *Allanblackia floribunda* Seed Extract Attenuates the Ethanol-Induced Gastric Ulcer in Rats via the Inhibition of TNF-*α* and INF-*γ* Levels and Modulation in the Expression of Ki67 Protein

**DOI:** 10.1155/2021/6694572

**Published:** 2021-01-11

**Authors:** Francis Ackah Armah, Isaac Tabiri Henneh, John Alake, Wisdom Ahlidja, Benjamin Amoani, Eric Gyamerah Ofori, Baffour Asante-Kyei, Gbadamosi Ismail Temitayo, Christian Kweku Adokoh

**Affiliations:** ^1^Department of Biomedical Sciences, School of Allied Health Sciences, College of Health and Allied Sciences, University of Cape Coast, Cape Coast, Ghana; ^2^Department of Pharmacology and Toxicology, School of Pharmacy and Pharmaceutical Sciences, College of Health and Allied Sciences, University of Cape Coast, Cape Coast, Ghana; ^3^Department of Biochemistry, School of Biological Sciences, College of Agriculture and Natural Sciences, University of Cape Coast, Cape Coast, Ghana; ^4^Division of Neurobiology, Department of Anatomy, Faculty of Basic Medical Sciences, College of Health Sciences, University of Ilorin, Ilorin, Nigeria; ^5^Department of Forensic Sciences, School of Biological Sciences, College of Agriculture and Natural Sciences, University of Cape Coast, Cape Coast, Ghana

## Abstract

*Allanblackia floribunda* has been used to treat an upset stomach in African traditional medicine, but its efficacy and safety have not been scientifically studied. The present research is aimed at assessing the antiulcer property of the seed extract of the plant to validate its traditional claim. Rats were pretreated with three doses of aqueous extract of *A*. *floribunda* (AFE) at 30, 100, and 300 mg/kg or omeprazole 10 mg/kg for 1 hr before the acute gastric ulcer was induced by oral administration of 5 mL/kg of 98% ethanol. The animals were sacrificed under anesthesia, and the stomach and blood were collected. The gross histology of the stomach, percentage protection conferred by the treatment, gastric pH, and serum TNF-*α* and INF-*γ* were assessed as well as the expression of Ki67 antigens. The antioxidant properties as well as the acute toxicity profile of the plant extract were also assessed. The results show that *A*. *floribunda* conferred significant protection on the rats against gastric ulceration with % protection of 46.15, 57.69, and 65.38 for AFE 30, 100, and 300 mg/kg, respectively, as well as 69.23% for omeprazole 10 mg/kg. The plant extract caused marked reductions in gastric pH, TNF-*α*, and INF-*γ* with statistical significance (*p* < 0.001) for AFE 300 mg/kg and omeprazole 10 mg/kg. Also, the plant showed good antioxidant activity comparable to gallic acid. Furthermore, the plant extract modulated the expression of Ki67 antigens. All animals survived the 14-day delayed toxicity test with no significant differences in physical, hematological, and biochemical parameters between rats orally administered with supratherapeutic doses of AFE (5000 mg/kg) or normal saline. The study established that the gastroprotective effect of the seed extract of *A*. *floribunda* is attributable to its antisecretory, antioxidant, and anti-inflammatory properties. Additionally, the plant was found to promote ulcer healing via the modulation of the expression Ki67 and was safe at supratherapeutic doses.

## 1. Introduction

Peptic ulceration occurs as a result of the erosion of the gastrointestinal mucosa that stretches through the muscularis mucosa deep into layers of the gut walls [1]. The disease has been a global threat for the past centuries, with a substantial effect on morbidity and mortality [2]. Risk factors for gastric ulcers include long-term use of steroidal and nonsteroidal anti-inflammatory drugs (NSAIDs), *Helicobacter pylori* infection, smoking, and alcohol intake [1, 3]. Alcohol, particularly, has been shown to elevate reactive oxygen species production and obstruct mitochondrial electron transport, leading to cell damage and gastric mucosal injury [4]. Also, alcohol triggers the release of other inflammatory mediators and cytokines such as TNF-*α* and INF-*γ*, which further aggravate gastric ulceration [4]. Adinortey et al. intimated that the ethanol-induced ulcer model resembles acute gastric ulcers in humans and it is particularly useful in assessing test agents with cytoprotective and antioxidant properties [5]. It is therefore a very useful model to employ in the assessment of prospective antiulcer agents. Already, drug classes such as proton pump inhibitors, histamine (H_2_) inhibitors, and antacids have been used to treat this disease for the past decades. However, these antiulcer agents have several shortcomings, such as impotency, arrhythmia, gynecomastia, arthralgia, and numerous drug-drug interactions, which make them unsafe for human use [6]. Additionally, the eradication of *H*. *pylori*-infested peptic ulcers remains a challenge resulting in the adoption of triple and quadruple therapies with their attendant problems of polypharmacy. This calls for the need to search for newer antiulcer agents that are safe and effective.

The therapeutic significance of medicinal plants has never been in doubt as many natural products from plants have served as potential treatments against several diseases, including gastric ulcers [7, 8]. It is not surprising that about 25% of therapeutic agents in orthodox practice for the past three decades originated from plants [9]. One plant genus that has been widely utilized in the traditional management of diverse diseases is the *Allanblackia*. The genus *Allanblackia* is a member of the flowering plant family *Clusiaceae*, which comprises 14 genera and almost 600 species of trees or shrubs that are mainly distributed in tropical regions of the world [10]. The genus extends across Africa from east of Nigeria to eastward of Uganda [10]. *Allanblackia floribunda*, a member of the genus, is an evergreen tree that grows up to 30 m tall with a trunk diameter of about 80 cm when mature [11]. It has a reddish-brown bark with small, irregular scales. The tree also has opposite, simple, entire, glabrous, estipulate leaves with short petioles. The flowers are unisexual, regular, and pinkish to reddish. It has large, ellipsoid, berry-like fruits with at least 40 and up to 100 seeds [10]. In English, it is called a tallow tree [11]. The fruit of *A*. *floribunda* is locally called “Uzoka” (Edo), “Orogbo-erin” (Yoruba), and “Egba” (Ibo) [10]. In Ghana, it is called “Okisidwe” by the Ashanti and “Sonyi” by the Nzema [12]. Traditionally, the decoction of the stem bark and leaves is used for treating dysentery, toothache, asthma, bronchitis, urethral discharge, and cough among the people of Gabon and the Democratic Republic of the Congo [13]. In Nigeria and Ghana, the plant is reported to relieve an upset stomach, body pains, hypertension, malaria, headache, and infertility [14]. Phytochemical analysis of the seed revealed the presence of alkaloids, phenols, saponins, tannins, and anthocyanins [15]. High-Performance Liquid Chromatography (HPLC) analysis has revealed the presence of flavonoids such as naringenin, eriodictyol, apigenin, and luteolin [16]. Even though the plant is being used traditionally to treat an upset stomach, this has not been scientifically authenticated, hence the need for the current research.

## 2. Materials and Methods

### 2.1. Drugs and Chemicals

Ethanol was obtained from the British Drug House (Poole, UK), whereas omeprazole, DPPH, Griess reagent, quercetin, gallic acid, diaminobenzidine tetrachloride, bovine serum albumin, and other reagents used were of analytical grade bought from Sigma-Aldrich Inc. (St. Louis, MO, USA). Mice Ki67 protein (code: sc-23900) was obtained from Santa Cruz Biotechnology (Santa Cruz, CA, USA). DuoSet ELISA reagents were obtained from R&D Systems Inc. (Minneapolis, USA).

### 2.2. Plant Material Collection

Fresh seeds of *Allanblackia floribunda* were collected from Axim (4°51′56.6^″^N; 2°13′51.0^″^W) in Nzema East District, Western Region, Ghana, in April 2020. The plant was identified and authenticated by a botanist at the Herbarium of School of Biological Sciences, University of Cape Coast, and a voucher specimen was preserved (ID No.: FAA/DR/008).

### 2.3. Preparation of the Extract

The seeds of *Allanblackia floribunda* were washed with water and air-dried for three weeks. The seeds were then pulverized using a hammer mill to obtain a fine powder. With the aid of a Soxhlet apparatus (Sigma-Aldrich Inc., St. Louis, MO, USA), 500 g of the powder was first defatted with petroleum ether (500 mL). The powder was then dried and subsequently extracted with distilled water (1 L) by cold maceration. The resulting filtrate from the aqueous extraction was concentrated using a rotary evaporator at a reduced temperature of 40°C. The extract was further dried in a desiccator containing activated silica to obtain a solid brown extract (AFE, 18.5 g). It was subsequently stored in a refrigerator, and the required doses were reconstituted immediately before drug administration.

### 2.4. Phytochemical Screening

The qualitative phytochemical test was performed on the *Allanblackia floribunda* powder according to methods described by Richardson and Harborne [17].

### 2.5. Total Phenolic and Flavonoid Content

#### 2.5.1. Total Phenolic Content (TPC) of AFE

The total phenolic content of AFE was determined according to the Folin-Ciocalteu method as described by Wolfe et al. [18] with slight modifications. The reaction mixture comprised 125 *μ*L of a dilute concentration of the plant extract (1 mg/mL), 375 *μ*L of the Folin-Ciocalteu reagent (prepared to a final dilution of 1 in 10), and 375 *μ*L of 7% Na_2_CO_3,_ which was added after incubating the initial mixture for 5 min. The reaction mixture was then made to 2.5 mL with distilled water and incubated at room temperature for 2 h for color development. The total phenolic content in gallic acid equivalence (GAE) was obtained spectrophotometrically (PG Instruments T70, Leicestershire, UK) by measuring the optical density at 765 nm against a reaction blank. A standard curve of gallic acid was prepared in the range of 0–300 *μ*g/mL. The experiment was repeated three times at each concentration.

#### 2.5.2. Total Flavonoid Concentration of AFE

The total flavonoid content was estimated by the aluminum trichloride colorimetric method using quercetin as the standard according to the original procedure described by Zhishen et al. [19] with few modifications. Exactly 200 *μ*L of AFE (1 mg/mL) was added to 800 mL of 50% ethanol. To this, 60 *μ*L of 5% sodium nitrite was added and incubated at room temperature for 5 min. Afterward, 60 *μ*L of 10% aluminum chloride was added and then incubated for a further 6 min before adding 400 *μ*L of 1 M sodium hydroxide. The resultant mixture was immediately diluted with 660 *μ*L of distilled water and thoroughly mixed. The colored complex formed was then measured at 510 nm. Varying concentrations of quercetin in ethanol were used as the standard, and the estimated total flavonoid content was expressed in quercetin equivalence (QE).

### 2.6. *In Vitro* Antioxidant Activity of AFE

#### 2.6.1. Free Radical Scavenging Assay by the Use of the DPPH Radical

The measurement of the scavenging activity of AFE against a stable 2,2-diphenyl-1-picrylhydrazyl (DPPH) radical was done using a previously established method [20]. The DPPH decolorizing potential of AFE was obtained by measuring the residual DPPH optical density at 517 nm after incubating the reaction mixture in the dark for 30 min. The purple/violet DPPH fades to a yellow color in the presence of substances with antioxidant properties. The reaction was initiated by adding the methanolic solution of DPPH (0.5 mM, 1 mL) to 1 mL of various concentrations (0-1500 *μ*g/mL) of the AFE and standard gallic acid, which was also prepared in methanol. A control setup consisting of an equal volume of DPPH and methanol was prepared. All reaction mixtures were thoroughly mixed before incubating them at room temperature in the dark. The percentage of radical activity was calculated from the absorbances obtained using the following equation:

(1) $$\begin{array}{c} \%\text{inhibition}=\frac{\text{Ac}-\text{At}}{\text{Ac}}\times 100, \end{array}$$

where Ac is the absorbance of the control and At is the absorbance of the test (extract/standard).

A graph of the percentage of scavenging activity against AFE/standard concentration in *μ*g/mL was plotted from which IC_50_ values were calculated. All experiments were conducted in triplicate, and results were reported in mean ± standard error of the mean.

#### 2.6.2. Nitric Oxide Scavenging Assay

The nitric oxide scavenging ability of AFE was estimated using the Griess-Ilosvay reaction [21]. The Griess-Ilosvay reagent utilizes 0.1% (*w*/*v*) naphthyl ethylenediamine dihydrochloride. AFE, ascorbic acid, and gallic acid at concentrations (0-1500 *μ*g/mL) were each prepared to 0.25 mL into separate test tubes. Then, 0.5 mL of 10 mM sodium nitroprusside was added, followed by the addition of 0.125 mL of the sodium phosphate buffer (pH 7.4). The reaction mixtures were then incubated in the dark at 25°C for 180 min. Afterward, 0.25 mL of the sulfanilic acid reagent (0.33% in 20% glacial acetic acid) was added and allowed to stand for 5 min for the completion of the reaction of diazotization. The completion of the reaction was achieved by adding to the mixture 0.25 *μ*L of 0.1% naphthyl ethylenediamine dihydrochloride to form a pink-colored solution. All test tube contents were well mixed and allowed to stand at 25°C for a further 30 min. The nitrite concentration was evaluated at 546 nm by preparing a control setup, which had everything except the replacement of the extract/standard volume with a buffer. The percentage NO^−^ scavenging abilities were calculated as follows:

(2) $$\begin{array}{c} \%\text{inhibition}=\frac{\text{Ac}-\text{At}}{\text{Ac}}\times 100, \end{array}$$

where Ac is the absorbance of the control and At is the absorbance of the test (extract/standard).

A graph of the percentage of inhibition against concentration in *μ*g/mL was plotted from which IC_50_ values were calculated. All experiments were conducted in triplicate, and results were reported in mean ± standard error of the mean.

#### 2.6.3. Reduction of Ferric Ion Assay

The reduction of ferric ions by the extract was determined using the *ortho*-phenanthroline procedure [22]. To each test tube containing varying concentrations (0-1500 *μ*g/mL) of the extract/standard, 0.5 mL of *o*-phenanthroline (in methanol) was added, followed by 1 mL of 200 *μ*M FeCl_3_. After incubating the mixture at room temperature for 10 min, the absorbance at 510 nm was measured using a spectrophotometer. Gallic acid and ascorbic acid were used as standards, and a graph of absorbance against concentration was plotted for analysis to obtain EC_50_ values.

### 2.7. Experimental Animals

Forty (40) male Sprague-Dawley rats (8-10 weeks old, 180-220 g), used in this experiment, were purchased from Noguchi Memorial Institute for Medical Research (NMIMR), Ghana. The animals were kept at the animal research facility of the Department of Biomedical Sciences, University of Cape Coast (UCC). They were allowed to acclimatize with the laboratory conditions for 15 days under standard conditions of room temperature (25 ± 1°C) and relative humidity (40 ± 10%) with a 12/12 hrs light/dark cycle before the experiments. The animals were secured in square stainless steel cages with softwood shaving as bedding, cleaned and maintained daily, and fed with standard commercial pelleted rodent feed (Flour Mills of Ghana Limited, Tema, Ghana) and water *ad libitum* during acclimatization and experimental periods. The National Institute of Health Guidelines for the Care and Use of Laboratory Animals were adhered to throughout the study. All procedures employed in this study were approved by the University of Cape Coast Ethical Review Board.

### 2.8. Acute Toxicity of AFE

Two groups (*n* = 5) of male Sprague-Dawley rats (8-10 weeks old, 180-220 g) were acclimatized for 15 days before the commencement of the test. A single dose of 5000 mg/kg was administered orally with an oral gavage to one group, and the other group received distilled water (10 mL/kg body weight). The animals were observed for clinical signs of toxicity at 10, 30, 60, and 120 min and at 4, 6, 12, and then 24 hrs after dosing. After 24 hrs, the animals were observed daily for 14 days. On the fifteenth day, all the animals were humanely euthanized under light anesthesia. Blood was collected from the heart for hematological and biochemical analyses.

### 2.9. Assessment of Antiulcer Activity of AFE

#### 2.9.1. Ethanol-Induced Ulcer

The test was performed using earlier described protocols [23–25] with slight modifications. Animals fasted for 24 hrs before the administration of test agents. The fasted animals were pretreated with AFE (30, 100, and 300 mg/kg, *p*.*o*.), omeprazole (10 mg/kg, *p*.*o*.), or normal saline (10 mL/kg, *p*.*o*.). Sixty (60) min after the treatment, the ulcer was induced via oral administration of 5 mL/kg of 98% ethanol. The animals were humanely sacrificed under diethyl ether anesthesia 60 min after ulcer induction.

#### 2.9.2. Determination of the Effect of AFE on Gastric pH

The content of the stomach was washed with 10 mL of normal saline into plain test tubes and centrifuged at 4000 rpm for 10 min. The pH of the supernatant was measured using a Digital pH Meter (MODEL # 152–R, Reena Instruments Company, New Delhi, India).

#### 2.9.3. Determination of the Effect of AFE on the Ulcer Index

The stomach was dissected along the greater curvature, washed, and gently stretched on a white sheet of paper. Gross examination of the stomach was carried out to assess the degree of ulceration by looking out for lesions, hemorrhages, erosions, and thickening of the gastric epithelia. The ulcer index was determined using the earlier described criteria [26] as follows: 0 = no lesion, 0.5 = hemorrhage, 1 = 1–3 small lesions < 10 mm length, 2 = 1–3 large lesions > 10 mm length, 3 = 1–3 thickened lesions, 4 = more than 3 small lesions, 5 = more than 3 large lesions, and 6 = more than 3 thickened lesions. The ulcer index (UI) was then used to calculate the percentage protection of the treatments by the following formula:

(3) $$\begin{array}{c} \%\text{protection}=\frac{\text{mean UI }\left(\text{untreated}\right)-\text{mean UI }\left(\text{treated}\right)}{\text{mean UI }\left(\text{untreated}\right)}\times 100\%. \end{array}$$

#### 2.9.4. Histological Analysis of the Gastric Tissues

The stomach was fixed in 10% phosphate-buffered formalin for 24 hrs. Portions of the fixed tissues were processed for routine histopathology, embedded in paraffin, sectioned at 5 *μ*m, and stained in hematoxylin and eosin [27]. Sections were examined using an Olympus microscope mounted with a live view digital SLR camera (E-330), and photomicrographs were taken.

#### 2.9.5. Immunohistochemical Analysis (Ki67 Expression)

Formalin-fixed paraffin-embedded tissue (3-4 micron) was cut and mounted on positively charged slides and air-dried for 20 min at a temperature of 80°C. Afterward, the tissue was deparaffinized (using 3 changes of xylene), dehydrated (using 5 changes of alcohol of increasing concentration), and rehydrated (using distilled water). Heat-induced antigen retrieval was performed using citrate buffer solution at 98°C to reveal a masked epitope. Subsequently, nonspecific protein reaction blocking was performed in 10% normal goat serum in 10 mM PBS+0.03% Triton X-100 and 1% bovine serum albumin (BSA) for 2 hrs at room temperature. The endogenous peroxidase block was done using 0.3% hydrogen peroxide in TBS (15 min). Sections were then incubated in 500 *μ*L primary monoclonal anti-mouse antibody for Ki67 protein (code: sc-23900, dilution 1 : 200) (Santa Cruz Biotechnology, Santa Cruz, CA, USA), diluted in the blocking buffer overnight at 4°C. After adequate washes, appropriate HRP-conjugated secondary antibodies were diluted in TBS+1% BSA and applied to slides for an incubation period of 1 hr at room temperature. The immunogenic reaction was developed using 3′3′ diaminobenzidine tetrachloride (DAB). Sections were then counterstained in hematoxylin, washed, dehydrated in absolute alcohol, cleared in xylene, and mounted in DPX. Cells immunoreactive for Ki67 were detected by the presence of a dark reddish-brown chromogen in the nucleus and quantified using the public domain software ImageJ.

#### 2.9.6. Assessment of AFE's Effect on Serum TNF-*α* and INF-*γ* Levels in the Ethanol-Induced Ulcer

About 3 mL of blood was collected by venipuncture of the heart into Vacutainer Hemogard serum separator tubes (SST) (Becton, Dickinson and Company, Temse, Belgium). Separation of serum in SST was accomplished by centrifugation at 4000 rpm for 5 min. Serum was collected and stored at -80°C until they were used for the cytokine assays. Serum levels of tumor necrosis factor-alpha (TNF-*α*) and interferon-gamma (INF-*γ*) were determined using the DuoSet ELISA reagents (R&D Systems Inc., Minneapolis, USA) following the manufacturer's instructions.

### 2.10. Statistical Analysis

Data has been presented as mean ± standard error of the mean (SEM). GraphPad® Prism Version 7.0 (GraphPad Software, San Diego, CA, USA) for Windows was used to perform all statistical analyses with *p* < 0.05 considered statistically significant for all tests. Differences between treatment groups were determined using one-way analysis of variance (ANOVA) followed by Dunnett's multiple comparison test.

## 3. Results

### 3.1. Phytochemical Analysis

*Allanblackia floribunda* aqueous seed extract was found to contain alkaloids, triterpenoids, steroids, phenols, saponins, and reducing sugars (Table 1).

### 3.2. Total Phenolic and Flavonoid Content

**Figure 1:**
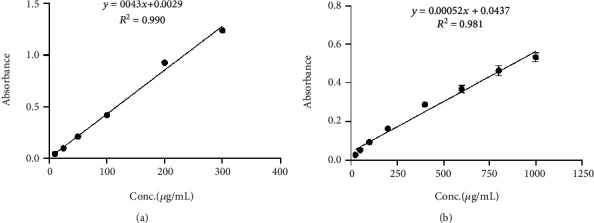
Standard graphs of (a) gallic acid for interpolation of the unknown phenolic content of extracts and (b) quercetin for interpolation of the unknown flavonoid content of extracts.

The total phenolic and flavonoid contents of AFE in gallic acid equivalence (GAE) and quercetin equivalence (QE) were found to be 30.605 GAE and 14.045 QE, respectively. These were obtained using the standard graphs for gallic acid and quercetin (Figure 1).

### 3.3. *In Vitro* Antioxidant Activity of AFE

#### 3.3.1. DPPH Scavenging Activity of AFE

**Figure 2:**
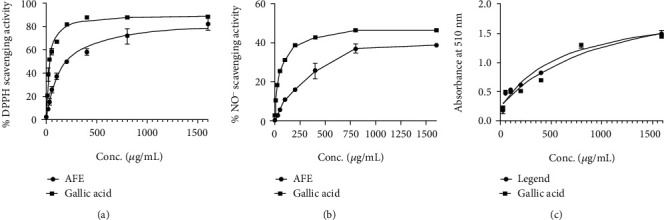
Graphs showing the (a) percentage DPPH scavenging capacity, (b) percentage nitric oxide radical scavenging activity, and (c) iron chelating ability of AFE and standard gallic acid at varying concentrations.

AFE exhibited a significant DPPH scavenging activity with IC_50_ of 146.9 *μ*g/mL, whereas gallic acid used as a positive control had IC_50_ of 28.19 *μ*g/mL (Figure 2(a)).

#### 3.3.2. Nitric Oxide Scavenging Activity of AFE

AFE showed a lesser nitric oxide scavenging activity with an IC_50_ = 218.1 *μ*g/mL compared to the gallic acid control, which had an IC_50_ of 43.18 *μ*g/mL (Figure 2(b)).

#### 3.3.3. Iron Chelating Abilities of AFE

AFE produced a higher ferric reducing power with an EC_50_ of 632.0 *μ*g/mL as compared to 1155 *μ*g/mL for the gallic acid control (Figure 2(c)).

### 3.4. Acute Toxicity Evaluation of AFE

No clinical signs of toxicity or death were observed up to 14 days following the administration of a supratherapeutic dose of 5000 mg/kg AFE orally to the experimental rats. Also, hematological and serum biochemical parameters were not significantly altered in the AFE treatment group compared to the naïve control rats (Tables 2 and 3).

### 3.5. Gross Anatomy and Histological and Immunohistochemical Analyses of the Protective Effect of AFE in the Ethanol-Induced Ulcer

**Figure 3:**
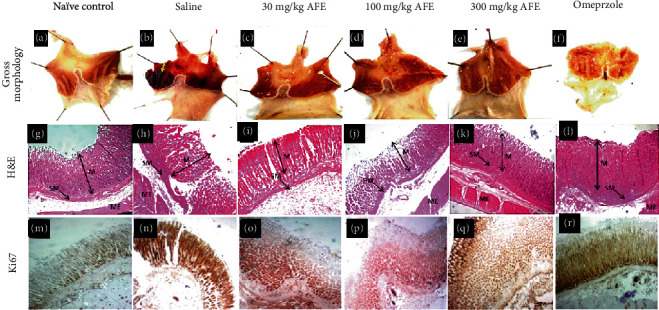
Representative gross morphology of the gastric lining of the stomach of experimental animals treated with saline (b), 30 mg/kg (c), 100 mg/kg (d), 300 mg/kg (e), or omeprazole (f) 60 min before the induction of the ulcer with 5 mL/kg of 98% ethanol. The naïve control group (a) presented with characteristically normal gastric morphology with a normal coloration of the intestinal lining. Group (b) presented with severe gastric ulceration as revealed by the eroded gastric lining and hemorrhagic manifestations (yellow arrow). Groups (c) and (d) presented with reduced ulceration and with lesser hemorrhage relative to group (b). Group (f) showed no hemorrhage but mild erosion at some parts of the organ. The section presents photomicrographs of the stomachs showing a panoramic view of the histomorphology of the gastric wall of experimental animals treated with saline (h), 30 mg/kg (i), 100 mg/kg (j), 300 mg/kg (k), or omeprazole (l) versus a naïve control (g). The micrographs presented with delineation of the gastric wall showing the layers of the stomach, mucosa (M), submucosa (SM), and muscularis externa (ME). Group (g) presented with the mucosal lining that is characterized by the intact epithelial lining, typical staining intensity, well-defined cellularity, and no pathological alterations. Groups (h) and (i) presented with distorted gastric histology with the eroded gastric lining and poorly defined submucosal layer. Groups (j)–(l) present with gastric histomorphology that looks characteristically normal. Their staining intensity and histomorphological delineation reveal no apparent histopathological alterations. The third section represents photomicrographs of the stomachs showing an immunohistochemical demonstration of Ki67 in the gastric mucosa of experimental animals treated with saline (n), 30 mg/kg (o), 100 mg/kg (p), 300 mg/kg (q), or omeprazole (r) versus a naïve control (m). Magnification for H&E and Ki67 staining was ×40.

Unlike the naïve control group (Figure 3(a)), which showed normal stomach architecture with no ulcerations, the gross appearance of the stomach of rats in the negative control group (Figure 3(b)) showed extensive ulceration and hemorrhage. There were progressively milder ulcerations in the groups treated with AFE 30, 100, and 300 mg/kg as well as omeprazole 10 mg/kg (Figures 3(c)–3(f)). The average ulcer index of the negative control group was 5.4, whereas the administration of AFE 30, 100, and 300 mg/kg markedly reduced the ulcer indices to averages of 2.8, 2.2, and 1.8, respectively. The standard drug omeprazole had an average ulcer index of 1.6 (Table 4).

Again, AFE significantly increased the pH of the stomach when compared with the negative control group (Table 4). The negative control group had an average pH of 4.6 whereas AFE 30, 100, and 300 mg/kg and omeprazole 10 mg/kg produced pHs of 6.29, 6.02, 6.56, and 6.52, respectively (Table 4).

To assess the protective effect of the extract on ulcerations induced in the stomach lining, sections of the stomach were stained with H&E for light microscopy. Figure 3(g) shows extensive damage to the mucosal lining in the group treated with saline one hour before the induction of the ulcer. The topmost part of the lining was eroded, and there were large lesions and tears in the negative control group (Figure 3(h)) compared to the naïve control animals (Figure 3(g)). The AFE 30 mg/kg group (C_1_)showed significant tissue distortion with mild tears and edema and eroded the top layer of the lining. AFE 100 (D_1_)AFE 300 mg/kg (E_1_)and omeprazole 10 mg/kg (F_1_)showed negligible damage to the tissue architecture.

High Ki67 activity was observed in the negative control group (Figure 3(n)) with a mean gray area of 123.1 ± 2.54 compared to 108.2 ± 2.86 seen in the naïve control group (Table 4). Treatment with 30, 100, and 300 mg/kg AFE significantly (*p* < 0.05 or *p* < 0.01) reduced the Ki67 activity to 108.6 ± 2.32, 103.7 ± 2.75, and 110.3 ± 2.28, respectively (Figures 3(o)–3(q) and Table 4). Although omeprazole treatment also substantially reduced the Ki67 activity to 109.6 ± 6.81, this was not statistically insignificant (Figure 3(r) and Table 4).

### 3.6. Effect AFE on TNF-*α* and INF-*γ* in the Ethanol-Induced Ulcer

**Figure 4:**
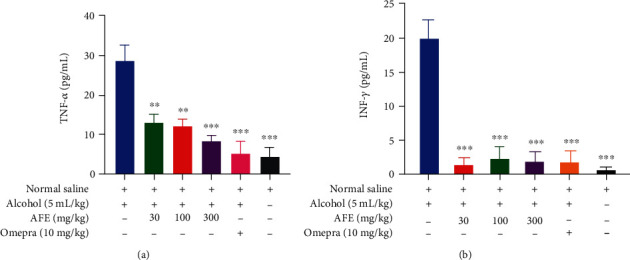
The effect of pretreatment of rats with AFE (30, 100, and 300 mg/kg), 10 mg/kg omeprazole, or 10 mL/kg saline before the induction of the gastric ulcer with 5 mL/kg of 98% ethanol on serum (a) TNF-*α* and (b) INF-*γ* levels. Data has been presented as mean ± standard error of the mean. The symbols ^∗∗^ or ^∗∗∗^ denote statistical significance at *p* < 0.01 or *p* < 0.001, respectively (one-way ANOVA followed by Dunnett's multiple comparison test).

The administration of 5 mL/kg of 98% *v*/*v* ethanol resulted in increased levels of both TNF-*α* and INF-*γ*. However, pretreatment of rats with AFE (30, 100, and 300 mg/kg) significantly (*p* < 0.01, *p* < 0.01, or *p* < 0.001, respectively) reduced the rats' serum TNF-*α* activity compared to the negative control group. The standard reference drug, omeprazole, also caused a significant (*p* < 0.001) reduction in the levels of TNF-*α* compared to the negative control group (Figure 4(a)).

Similarly, the treatment of rats with all doses of AFE and omeprazole produced a significant (*p* < 0.001) reduction in the levels of INF-*γ* compared to the negative control group (Figure 4(b)).

## 4. Discussion

Ethanol has been one of the most widely used *in vivo* animal models for the study of the gastric ulcer [24, 28]. High concentrations of ethanol cause rapid hyperemia, hemorrhage, and necrosis in the gastric mucosa [25]. Ethanol in the stomach is also associated with an increase in vasoactive molecules (such as histamine and leukotriene C4), mast cell degranulation, blood stasis, and elevation in gastric acid secretion within the first 60 min of exposure [28, 29]. This is consistent with the results of our study, which revealed extensive disruption of gastric mucosa, hemorrhage, and necrosis, resulting in a high ulcer index in the negative control group. These features were absent in the naïve control groups.

In the present study, treatment of rats with AFE resulted in a significantly reduced ulcer index with a corresponding increase in gastric pH compared to the diseased controls. The results obtained for AFE was comparable to that for omeprazole, a first-line drug used in the treatment of the gastric ulcer. Omeprazole, a proton pump inhibitor, exerts its gastroprotective and antisecretory effect via the inhibition of the H^+^/K^+^ ATPase pump [30]. Similar observations were made in the gross analysis of the stomach of animals pretreated with the extract (Figure 3). It can be inferred that AFE exhibited an antiulcer effect similar to omeprazole, the reference drug.

Ethanol is known to induce the release of free radicals, which causes oxidative stress and a subsequent increase in inflammatory response leading to gastric mucosal injury and ulcers [31]. Potent antioxidant agents have been shown to prevent tissue necrosis and ulcers induced by oxidative stress and its associated excessive inflammatory response [32]. In this present study, *A*. *floribunda* seed extract exhibited a significant *in vitro* antioxidant activity, which included higher ferric reducing power as well as DPPH and nitric oxide scavenging activities. This corroborates with previous *in vivo* and *in vitro* antioxidant assessment of the plant [14, 33]. This antioxidant property of the extract may have contributed to the antiulcer effect of the extract, as observed in the study. The control drug, omeprazole, has also been reported to have antioxidant activity [32], which underscores the importance of antioxidant properties in the healing of peptic ulcers.

Again, AFE at all doses significantly reduced the leve1s of inflammatory cytokines TNF-*α* and interferon-*γ* (Figure 4). The results obtained from the study are in sync with earlier findings, which indicate that administration of ulcerogenic substances significantly increases the levels of proinflammatory cytokines such as TNF-*α* and IFN-*γ* [34]. Importantly, of all the proinflammatory cytokines, TNF-*α* retains diverse roles in the pathogenesis of gastric ulcers, including activation of neutrophil infiltration, apoptosis, NF-*κ*B, and inducible nitric oxide synthase (iNOS) [33]. IFN-*γ*, on the other hand, exerts its proinflammatory activities via the stimulation of macrophages to produce an important proinflammatory cytokine IL-12 while inhibiting the proliferation of Th2-derived anti-inflammatory cytokines IL-4 and IL-10 [35]. The reduction in the levels of both TNF-*α* and IFN-*γ* by the extract suggests its gastroprotective and anti-inflammatory property is mediated by its inhibitory effect on those cytokines. This also corroborates with an earlier report on the anti-inflammatory effect of the plant [13].

Ki67 is a nuclear matrix protein expressed in a dividing cell but not in quiescent cells [36]. It indicates an elevated cell proliferation rate, which is considered an important predictive and prognostic marker in several cancers, including gastrointestinal stromal tumors [37, 38]. This research found a strong Ki67 activity (123.1 ± 2.54 of the mean gray area) in the diseased controls compared to the naïve controls (108.2 ± 2.86), as presented in Figure 3 and Table 4. However, pretreatment of rats with AFE before the induction of the ulcer resulted in a significant (*p* < 0.05) reduction in the expression of Ki67. This suggests that the extract offers a modulatory effect on the cell proliferative activity of ethanol in addition to its gastroprotective effect. This is consistent with earlier studies, which suggest that the antiulcerogenic capacity of a plant extract was accompanied by a modulatory effect in the expression of Ki67 protein [32].

The acute toxicity profile in this study showed no negative effect, and neither has there been any report of the deleterious effect associated with the use of *A*. *floribunda* despite its extensive use for several therapeutic purposes in various communities. The administration of therapeutic doses to animals for a long period is currently ongoing in our laboratories to ascertain its chronic toxicity profile.

The seed extract of *A*. *floribunda* was found to contain secondary metabolites such as flavonoids, alkaloids, terpenoids, fatty acids, and saponins among others. This agrees with earlier reports on the plant [16, 39], which suggests that flavonoids and phenolic compounds possess good antiulcer and anti-inflammatory properties alongside other phytochemicals [33, 40]. It is suggested that the presence of those phytochemicals contributed to the amelioration of gastric ulcers induced by ethanol administration.

## 5. Conclusion

The current study establishes the gastroprotective effect of the seed extract of *A*. *floribunda* attributable to its antisecretory, antioxidant, and anti-inflammatory properties. Additionally, the plant was found to promote ulcer healing via the modulation in the expression of Ki67 protein and was found to be safe at supratherapeutic doses. Further research on the isolation of the active compounds responsible for this effect is currently ongoing in our laboratories.

## Figures and Tables

**Table 1:** Phytochemical analysis of aqueous seeds of *Allanblackia floribunda*.

Constituents	Present/absent
Alkaloids	+
Terpenoids	+
Fatty acids	+
Reducing sugars	+
Phenols	+
Saponins	+
Steroids	+
Flavonoids	+

The symbols (+) and (-) indicate present and absent, respectively.

**Table 2:** Serum biochemical parameters of rats treated with a single dose of 5000 mg/kg AFE or normal saline.

Parameter	AFE 5000 mg/kg	Normal saline
Albumin (g/L)	34.27 ± 0.50	33.55 ± 0.98
Total protein (g/L)	65.36 ± 1.43	65.30 ± 2.63
Globulin (g/L)	31.13 ± 0.92	31.74 ± 1.87
Total bilirubin (*μ*mol/L)	2.17 ± 0.05	3.89 ± 0.09
D. bilirubin (*μ*mol/L)	1.40 ± 0.15	1.48 ± 0.15
Ind. bilirubin (*μ*mol/L)	0.77 ± 0.19	2.41 ± 0.14
AST (U/L)	176.80 ± 19.46	202.93 ± 17.59
ALP (U/L)	550.27 ± 98.85	437.30 ± 63.96
ALT (U/L)	62.70 ± 7.22	45.08 ± 0.93
GGT (U/L)	0.38 ± 0.26	0.30 ± 0.18
Creatinine (*μ*mol/L)	54.03 ± 6.15	67.63 ± 4.28
Urea (mmol/L)	3.86 ± 0.34	15.23 ± 12.16
BUN/CRE	74.41 ± 13.55	55.80 ± 11.03

Results are presented as mean ± SEM. One-way ANOVA revealed no statistical significance between the treatment and control groups (*p* > 0.05, *n* = 5). ALT: alanine aminotransferase; ALP: alkaline phosphatase; AST: aspartate aminotransferase; BUN/CRE: blood urea nitrogen/creatinine.

**Table 3:** Hematological parameters of rats treated with a single dose of 5000 mg/kg AFE or normal saline.

Hematological parameter	AFE 5000 mg/kg	Normal saline
WBC (10^9^/L)	9.34 ± 1.13	9.08 ± 1.13
RBC (10^12^/L)	6.87 ± 0.47	6.58 ± 0.32
HGB (g/dL)	12.37 ± 0.21	13.07 ± 0.82
HCT (%)	37.90 ± 2.41	37.62 ± 1.62
MCV (%)	55.17 ± 0.67	55.44 ± 0.74
MCH (pg)	18.00 ± 0.26	17.90 ± 0.32
MCHC (g/dL)	32.63 ± 0.09	32.33 ± 0.12
PLT (10^9^/L)	644.33 ± 52.92	651.33 ± 82.87
PDW (fL)	7.90 ± 0.50	7.75 ± 0.45
MPV (fL)	7.87 ± 0.28	7.92 ± 0.16
P_LCR	9.17 ± 2.38	8.87 ± 2.44
PCT (%)	0.51 ± 0.05	0.71 ± 0.02

Results are presented as mean ± SEM, *p* < 0.05 (*n* = 5). WBC: white blood cell count; RBC: red blood cell count; HGB: hemoglobin; HCT: hematocrit; MCH: mean corpuscular hemoglobin; MCHC: mean corpuscular hemoglobin concentration; MCV: mean corpuscular volume; PLT: platelet count; MPV: mean platelet volume; PDW: platelet distribution width; PCT: plateletcrit.

**Table 4:** Average ulcer index, and percentage (%) protection, gastric pH, and Estimated mean gray area of aqueous seed extract of *Allanblackia floribunda* (30, 100, and 300 mg/kg) or omeprazole in the ethanol-induced ulcer.

Treatment	Ulcer index	Average protection (%)	Average pH	Est. mean gray area^#^
Naïve control (10 mL/kg saline)	—	—	5.22 ± 0.28	108.2 ± 2.86^∗^
Alcohol+10 mL/kg saline	5.2 ± 0.37	—	4.62 ± 0.21	123.1 ± 2.54
Alcohol+30 mg/kg AFE	2.8 ± 0.37^∗∗∗^	46.15	6.2 ± 0.46^∗∗∗^	108.6 ± 2.32^∗^
Alcohol+100 mg/kg AFE	2.2 ± 0.37^∗∗∗^	57.69	6.02 ± 0.18^∗∗^	103.7 ± 2.75^∗∗^
Alcohol+300 mg/kg AFE	1.8 ± 0.37^∗∗∗^	65.38	6.56 ± 0.40^∗∗∗^	110.3 ± 2.28^∗^
Alcohol+10 mg/kg omeprazole	1.6 ± 0.24^∗∗∗^	69.23	6.52 ± 0.13^∗∗∗^	109.6 ± 6.81

Data has been presented as group means (*n* = 5) ± standard error of means. The symbols ^∗^, ^∗∗^, or ^∗∗∗^ denote *p* < 0.05, *p* < 0.01, or *p* < 0.001, respectively, compared to the negative control (one-way ANOVA followed by Dunnett's multiple comparison test). ^#^Estimated mean gray of the Ki67 activity.

## Data Availability

All data generated or analyzed during this study are included in this manuscript.

## References

[1] Drina M. (2017). Peptic ulcer disease and non-steroidal anti-inflammatory drugs. *Australian Prescriber*.

[2] Malfertheiner P., Chan F. K. L., Mccoll K. E. L. (2009). Peptic ulcer disease. *Lancet*.

[3] Ramakrishnan K., Salinas R. C. (2007). Peptic ulcer disease. *American Family Physician*.

[4] Ma L., Dong J.-X., Wu C. (2017). Spectroscopic, polarographic, and microcalorimetric studies on mitochondrial dysfunction induced by ethanol. *The Journal of Membrane Biology*.

[5] Adinortey M. B., Ansah C., Galyuon I., Nyarko A. (2013). In vivo models used for evaluation of potential antigastroduodenal ulcer agents. *Ulcers*.

[6] Tripathy S., Afrin R. (2016). Herbal treatment alternatives for peptic ulcer disease. *Journal of Drug Delivery and Therapeutics*.

[7] Hussaini J. (2012). Gastroprotective effects of Dicranopteris linearis leaf extract against ethanol-induced gastric mucosal injury in rats. *Scientific Research and Essays*.

[8] AlRashdi A. S., Salama S. M., Alkiyumi S. S. (2012). Mechanisms of gastroprotective effects of ethanolic leaf extract of *Jasminum sambac* against HCl/ethanol-induced gastric mucosal injury in rats. *Evidence-Based Complementary and Alternative Medicine*.

[9] Li F., Wang Y., Li D., Chen Y., Dou Q. P. (2019). Are we seeing a resurgence in the use of natural products for new drug discovery?. *Expert Opinion on Drug Discovery*.

[10] Crockett S. (2015). Allanblackia oil: phytochemistry and use as a functional food. *International Journal of Molecular Sciences*.

[11] Orwa C., Mutua A., Kindt R., Jamnadass R. (2009). A tree reference and selection guide version 4.0. *Agroforestree Database*.

[12] Irvine F. (1961). *Woody Plant of Ghana*.

[13] Ayoola G. A., Akpanika G. A., Awobajo F. O., Sofidiya M. O., Osunkalu V. O., Odugbemi T. O. (2009). Anti-inflammatory properties of the fruits of Allanblanckia floribunda oliv. (Guttiferae). *Botany Research International*.

[14] Bilanda D. C., Dimo T., Djomeni P. D. D. (2010). Antihypertensive and antioxidant effects of *Allanblackia floribunda* Oliv. (Clusiaceae) aqueous extract in alcohol- and sucrose-induced hypertensive rats. *Journal of Ethnopharmacology*.

[15] Dike M. C. (2012). Proximate, phytochemical and mineral compositions of seeds of Allanblackia floribunda , Garcinia kola and Poga oleosa from Nigerian rainforest. *African Journal of Biotechnology*.

[16] Akpanika G. A., Winters A., Wilson T., Ayoola G. A., Adepoju-Bello A. A., Hauck B. (2017). Polyphenols from _Allanblackia floribunda_ seeds: identification, quantification and antioxidant activity. *Food Chemistrym*.

[17] Richardson P. M., Harborne J. B. (1990). Phytochemical Methods: A Guide to Modern Techniques of Plant Analysis. Second Edition. *Brittonia*.

[18] Wolfe K., Wu X., Liu R. H. (2003). Antioxidant activity of apple peels. *Journal of Agricultural and Food Chemistry*.

[19] Zhishen J., Mengcheng T., Jianming W. (1999). The determination of flavonoid contents in mulberry and their scavenging effects on superoxide radicals. *Food Chemistry*.

[20] Badmanaban R., Patel C. N., Patel V. (2010). Determination of Polyphenolic Content and In-vitro Antioxidant Capacity of the Leaves of _Lagenaria siceraria_ (mol.) standl. *Pharmacognosy Journal*.

[21] Patel A., Patel A., Patel N. M. (2010). Determination of polyphenols and free radical scavenging activity of Tephrosia purpurea Linn leaves (Leguminosae). *Pharmacognosy Research*.

[22] Afroze F., Hossain M. T. (2015). Proximate analysis, phytochemical screening and antioxidant activity of Psidium guajava leaves growing in coastal area of Bangladesh. *World Journal Pharmacy and Pharmaceutical Science*.

[23] Karampour N. S., Arzi A., Rezaie A., Pashmforoosh M., Kordi F. (2019). Gastroprotective effect of zingerone on ethanol-induced gastric ulcers in rats. *Medicina*.

[24] Fernandes H. B., Silva F. V., Passos F. F. B. (2010). Gastroprotective effect of the ethanolic extract of Parkia platycephala benth. Leaves against acute gastric lesion models in rodents. *Biological Research*.

[25] Oates P. J., Hakkinen J. P. (1988). Studies on the mechanism of ethanol-induced gastric damage in rats. *Gastroenterology*.

[26] Sofidiya M. O., Agufobi L., Akindele A. J., Olowe J. A., Familoni O. B. (2012). Effect of Flabellaria paniculata Cav. extracts on gastric ulcer in rats. *BMC Complementary and Alternative Medicine*.

[27] Slaoui M., Bauchet A. L., Fiette L. Tissue sampling and processing for histopathology evaluation. *Methods in Molecular Biology*.

[28] Aures D., Guth P. H., Paulsen G., Grossman M. I. (1982). Effect of increased gastric mucosal histamine on alcohol-induced gastric damage in rats. *Digestive Diseases and Sciences*.

[29] Jamal A., Javed K., Aslam M., Jafri M. A. (2006). Gastroprotective effect of cardamom, *Elettaria cardamomum* Maton. fruits in rats. *Journal of Ethnopharmacology*.

[30] Wallmark B. (2009). Mechanism of action of omeprazole. *Scandinavian Journal of Gastroenterology*.

[31] Park J. U., Kang J. H., Rahman M. A. A., Hussain A., Cho J. S., Lee Y. I. (2019). Gastroprotective effects of plants extracts on gastric mucosal injury in experimental Sprague-Dawley rats. *BioMed Research International*.

[32] Brito S. A., de Almeida C. L. F., de Santana T. I. (2018). Antiulcer Activity and Potential Mechanism of Action of the Leaves of Spondias mombin L.. *Oxidative Medicine and Cellular Longevity*.

[33] Boudjeko T., Megnekou R., Woguia A. L. (2015). Antioxidant and immunomodulatory properties of polysaccharides from Allanblackia floribunda Oliv stem bark and Chromolaena odorata (L.) King and H.E. Robins leaves. *BMC Research Notes*.

[34] Antonisamy P., Arasu M. V., Dhanasekaran M. (2016). Protective effects of trigonelline against indomethacin-induced gastric ulcer in rats and potential underlying mechanisms. *Food & Function*.

[35] Bae H., Barlow A., Young H., Valencia J. (2016). *Interferon γ: an overview of its functions in health and disease*.

[36] Asante M., Ahmed H., Patel P. (1997). Gastric mucosal hydrophobicity in duodenal ulceration: role of Helicobacter pylori infection density and mucus lipids. *Gastroenterology*.

[37] Basilio-De-Oliveira R. P., Pannain V. L. N. (2015). Prognostic angiogenic markers (endoglin, VEGF, CD31) and tumor cell proliferation (Ki67) for gastrointestinal stromal tumors. *World Journal of Gastroenterology*.

[38] Ma K., Baloch Z., He T. T., Xia X. (2017). Alcohol consumption and gastric cancer risk: a meta-analysis. *Medical Science Monitor*.

[39] Kuete V., Azebaze A. G. B., Mbaveng A. T. (2011). Antioxidant, antitumor and antimicrobial activities of the crude extract and compounds of the root bark of Allanblackia floribunda. *Pharmaceutical Biology*.

[40] de Almeida A. L., Beleza M. L. M. L., Campos A. (2017). Phytochemical profile and gastroprotective potential of Myrcianthes pungens fruits and leaves. *Nutrire*.

